# CD58 Immunobiology at a Glance

**DOI:** 10.3389/fimmu.2021.705260

**Published:** 2021-06-08

**Authors:** Yalu Zhang, Qiaofei Liu, Sen Yang, Quan Liao

**Affiliations:** Department of General Surgery, State Key Laboratory of Complex Severe and Rare Diseases, Peking Union Medical College Hospital, Chinese Academy of Medical Science and Peking Union Medical College, Beijing, China

**Keywords:** CD58, lymphocyte functional antigen-3, LFA-3, CD2, T cell activation, immune evasion

## Abstract

The glycoprotein CD58, also known as lymphocyte-function antigen 3 (LFA-3), is a costimulatory receptor distributed on a broad range of human tissue cells. Its natural ligand CD2 is primarily expressed on the surface of T/NK cells. The CD2-CD58 interaction is an important component of the immunological synapse (IS) that induces activation and proliferation of T/NK cells and triggers a series of intracellular signaling in T/NK cells and target cells, respectively, in addition to promoting cell adhesion and recognition. Furthermore, a soluble form of CD58 (sCD58) is also present in cellular supernatant *in vitro* and in local tissues *in vivo*. The sCD58 is involved in T/NK cell-mediated immune responses as an immunosuppressive factor by affecting CD2-CD58 interaction. Altered accumulation of sCD58 may lead to immunosuppression of T/NK cells in the tumor microenvironment, allowing sCD58 as a novel immunotherapeutic target. Recently, the crucial roles of costimulatory molecule CD58 in immunomodulation seem to be reattracting the interests of investigators. In particular, the CD2-CD58 interaction is involved in the regulation of antiviral responses, inflammatory responses in autoimmune diseases, immune rejection of transplantation, and immune evasion of tumor cells. In this review, we provide a comprehensive summary of CD58 immunobiology.

## Introduction

Intercellular adhesion is vital for a range of immunological responses, including the interaction between T lymphocytes and target cells. Conjugate formation of T cells with antigen-negative targets is nearly as efficient as with specific target cells without causing lysis of target cells. Therefore, on specific target cells, adhesion in an antigen-independent manner may occur simultaneously with or prior to antigen recognition (1).

**Figure 1:**
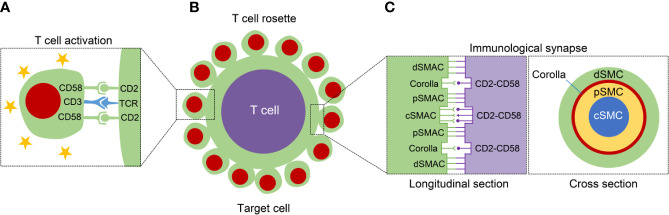
The structure diagram with regard to T cell activation, T cell rosette, and immunological synapse (IS). **(A)** The left panel displays that the CD2-CD58 interaction facilitates the T cell activation through offering the necessary second signal and assisting TCR-mediated stimulation. **(B)** The middle panel exhibits the formation of T cell rosette mainly mediated by the binding of CD2 with CD58. **(C)** The IS can be classified into different supramolecular activation complexes (SMAC), central, peripheral, and distal SMAC (c, p and dSMAC, respectively). In addition to the cSMAC, the CD2-CD58 interactions exist between pSMAC and dSMAC, and form a ring-like structure, called “corolla”. The right panel shows the longitudinal and cross section of IS.

The immune adhesion molecule CD58, also known as LFA-3, is a heavily glycosylated, distributed surface glycoprotein of 40-70 kDa and extensively expressed on hematopoietic and nonhematopoietic cells (2, 3). CD58 on the cell surface participates in potentiating effector-target adhesion during antigen-specific recognition (4). Cell-cell adhesion is crucial for leucocyte-mediated chemotaxis, phagocytosis, cytotoxicity, and induction of lymphocyte differentiation and proliferation. In terms of the antigen-presenting process, the CD58 molecule offers an effective second signal for T cell activation, thereby optimizing and replenishing the proliferative response mediated through TCR/CD3 signaling (**Figure 1A**) (5, 6).

CD2, also known as T11, LFA-2, the erythrocyte (E) rosette receptor, is the natural ligand of CD58. It is a surface glycoprotein restricted to T lymphocytes, NK cells, thymocytes, and a subset of bone marrow cells (7, 8). Both CD2 and CD58 are members of the immunoglobulin supergene family and their amino acid sequences on the extracellular domain are significantly similar (9). The amino-terminal domain of CD2 is responsible for target cell adhesion and binds to CD58 on target cells or antigen-presenting cells (APC) with high affinity (10–12). As an important adhesion pathway between T cells and target cells, CD2-CD58 interaction is not only a crucial costimulatory signal for optimal T cell activation in response to antigens, but also induction of a series of essential signal transduction events to participate in the modulation of T cell responses (13, 14). For example, incubation of B lymphoblastoid cell with immobilized anti-CD58 mAbs causes broad tyrosine phosphorylation and increases TNF-α production (15). Accumulating evidence has demonstrated that the CD2-CD58 interaction plays a critical role in lymphocyte activation, recirculation, and effector function, e.g., cytolytic activity on neoplastic cells (16, 17).

Herein, we have collated almost all of the published literature from discovery to the present and elaborately summarized the CD58 immunobiology in a systematic and comprehensive manner, including CD58 isoforms, sCD58, IS formation, CD58 polymorphisms, CD2-CD58 interaction, their structures of interface, and related functions; simultaneously dissected the important effects of CD58 for T/NK cell-mediated immune response in tumor-related and immune-related diseases.

## Two Isoforms of CD58

**Figure 2:**
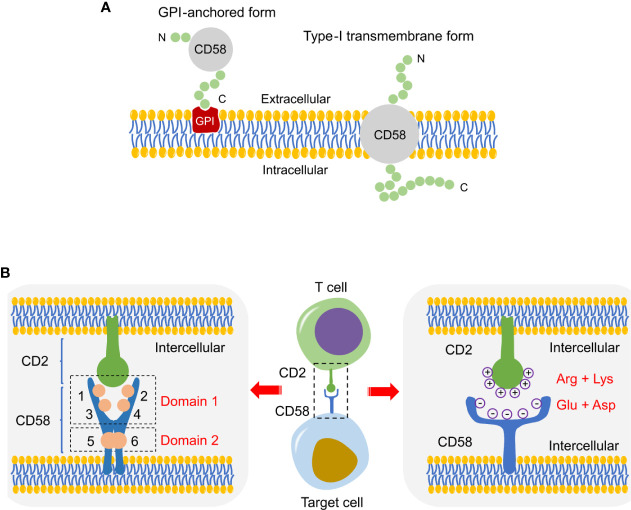
Schematic of CD58 isoforms and CD2-CD58 interface. **(A)** Schematic diagram of two CD58 isoforms, a GPI-anchored and a type-I transmembrane form. **(B)** Structure diagram of the interface in CD2-CD58, which is mainly supported by electrostatic complementarity instead of shape matching.

There are two isoforms of CD58 derived from divergent mRNA splicing: a type-I transmembrane and a glycosylphosphatidylinositol (GPI)-anchored form (**Figure 2A**) (18). The former has an extracellular domain with six N-linked glycosylation sites sequentially linked to a hydrophobic transmembrane region and a 12-amino acid cytoplasmic segment; The latter is anchored to the outer side of the cell membrane by a GPI tail without transmembrane region and cytoplasmic domain (18, 19). They are located in different membrane compartments. The GPI-anchored isoform resides in lipid raft, whereas the transmembrane isoform localizes in a non-raft microdomain (20). Despite the transmembrane CD58 outside lipid rafts, it can trigger signaling independently of the GPI-anchored isoform, such as induction of tyrosine phosphorylation of PLCγ, SYK and BLNK, and activation of AKT and ERK (20, 21). Cell adhesion is dependent on the density of CD58. At lower densities, GPI-linked isoform is crucial for enhancing adhesion, instead of the transmembrane isoform (22). Accordingly, unlike the well-accepted concept that the GPI-anchor is indispensable for signaling, the GPI-anchored CD58 is more effective in enhancing adhesion, whereas the transmembrane form is more critical for signal transduction. This kind of structural distribution is of great significance to CD58 adhesion and transmembrane signaling (23).

## CD2-CD58 Interaction

Human peripheral blood T lymphocytes have sheep red blood cells (SRBC) receptors on their surface. Human T lymphocytes are mixed with SRBC to form a rosette centered on T cells and surrounded by SRBCs *in vitro*, known as the “E-rosette test”, which reflects the immunological activity of T lymphocytes (**Figure 1B**). The formation of E-rosette is dependent on the binding of CD2 in T lymphocytes with T11 target structure (T11TS) on SRBC, which is a functionally homologous ligand for CD58 on human erythrocytes (24, 25). The anti-CD58 and anti-CD2 mAbs can inhibit rosette formation through acting on the erythrocyte and the T lymphocyte, respectively (26). In Hodgkin’s lymphoma (HL) tissue, spontaneous rosette formation of T cells with Reed-Sternberg cells is also mediated *via* CD2-CD58 interaction (26).

The interaction between CD2 in T cells and CD58 in target cells is subtle and unique. Activated human T lymphocytes can form rosettes with autologous erythrocytes, while resting T cells cannot (18, 27). Moreover, the interaction of CD2-CD58 is enthalpy-driven, accompanied by adverse entropic changes and energetically remarkable conformational adjustments (28). Unlike the other adhesion, CD2-CD58 interaction does not depend on cellular metabolism and cytoskeletal involvement, insensitive to ambient temperature, and its rate constant and average affinity not influenced by variations in ionic strength such as extracellular Mg^2+^/Ca^2+^ (26, 28).

## Structure of Interface in CD2-CD58

There are four discrete epitopes on the membrane-distal domain (domain 1) and two overlapping epitopes on the membrane-proximal domain (domain 2) in the CD58 molecule (**Figure 2B**) (29, 30). The N-terminus of the CD58 epitopes are functional sites involved in the interaction site with CD2, while domain 2 connects CD58 to membrane anchor independent of CD2 binding and CD58-mediated activation (29, 30). In addition, all epitopes exist in the same numbers on a wide variety of CD58-positive cells and show a uniform trend of increase/decrease after cell activation or malignant transformation (30). Protein-protein interface interactions are fundamentally supported by shape matching and electrostatic complementarity. The crystal structure between the binding interface of CD2 and CD58 is an orthogonal, asymmetric, and face-to-face interaction involving the main β-sheets of the respective N-terminal domains (31). The binding domain of CD58 is a localized and densely charged surface region on the AGFCC’C” face of the CD2 adhesion site (32). Through disrupting the highly acidic surface of the AGFCC’C” β-sheet of CD58, it was unexpectedly found that the CD2-CD58 interface lacks significant shape complementarity (33). The electrostatic potential on the CD2 surface is primarily positive because of arginine and lysine residues, whereas CD58 exhibits negative charge at the interface with CD2 due to the presence of glutamate and aspartate residues (**Figure 2B**) (34). Specifically, the CD2-CD58 binding site is composed of β-strands C and F with charged residues (34). Therefore, electrostatic attraction, rather than shape complementarity, plays a dominant role in the binding of CD2 to CD58 (35). This pattern of binding and recognition is strikingly different from the well-known interactions of other proteins, e.g., antibody-receptor or cytokine-cytokine receptor interactions (33). Under conditions of little hydrophobicity, the interlaced, charged amino acid side chains shape salt and hydrogen bonds at the interface, generating a high degree of specificity despite with low affinity that weaker than initial expectation, which satisfies the special requirements for such interaction to be easily reversible (36, 37). Selective binding, weak, reversible, these features are particularly suitable for CD2-CD58 interaction to initiate and sustain dynamic bindings between T/NK cells and target cells (36, 37). In addition, structural analysis shows that CD2-CD58 adhesion has strong conformational flexibility and some unnatural helical conformations under organic solvents or high-temperature conditions (38). The conformational state of the adhesion proteins is beneficial to the modulation of CD2 folding and cell adhesion (38).

As a costimulatory pathway, CD2-CD58 interactions provide a series of favorable conditions for signal recognition of T/NK cells with their targets. Firstly, substantial CD2-CD58 interactions contribute to overcoming intercellular charge repulsion, thereby eliminating bond strain on the interactions of TCR-ligand (39). Secondly, on account of the membrane gap of the CD2-CD58 complex is equal to that of the TCR peptide-MHC complex, numerous CD2-CD58 interactions would place the distance between T/NK cell and target cell within an optimal range for the T/NK cell receptor-ligand interaction (40). Thirdly, the cytoplasmic domain of CD2 is large and conserved, which facilitates the recruitment of cytoskeleton and signaling molecules into the contact cap (41–43).

Additionally, glycosylation plays a crucial role in intercellular adhesion and regulates the stability and dynamics of proteins in a subtle way (44), which is likewise involved in the regulation of human CD2/CD58-mediated cell-cell adhesion by conformational adjustment (45). Fully glycosylated CD58 is more effective in suppressing the formation of E-rosette than the deglycosylated form, so the maintenance of CD58 glycosylation is essential for the exertion of its functional activity (46). The CD2-CD58 interaction is largely administrated by three hot spots forming a binding triangle, the topology of which is fundamental for the stability of CD2-CD58 binding. The topology of CD2 conformation is remarkably tuned and induced by glycosylation into a specific structure to energetically stabilizes the CD2-CD58 complex. Therefore, CD2 glycosylation facilitates CD2-CD58 binding *via* conformational adjustment (45).

According to the relevant structure epitopes, drugs or agents are designed to influence CD2/CD58-mediated intercellular adhesion to regulate the immune response. In the CD2-CD58 interface, CD58 Lys34 and CD2 Tyr86 residues are functional hot spots (47). Therefore, short peptide drugs can be constructed from the hot spot β-strand area of CD2 molecule with CD58 binding site. For example, structural constraints from CD2 are inserted into the peptides *via* the dibenzofuran moiety to nucleate β-strand conformation in the peptides, thus regulating the binding of CD2 to CD58 (48). In the collagen-induced arthritis (CIA) mouse model, a peptidomimetic designed to disrupt the interface of CD2-CD58 interaction can inhibit the T/NK cell-mediated immune response through interfering with the binding of CD2 with CD58 (49). Besides, a kind of nonimmunogenic compound 7 is successfully synthesized to act as a lead compound for immunoregulation, accompanied by a reduction of IFN-γ and anti-collagen antibody levels in the CIA model, and thus it may be an effective therapeutic drug for the autoimmune disease (50). These results indicate that peptides targeting costimulatory molecule CD2/CD58 can be used to regulate immune responses and contribute to the development of therapeutic drugs for autoimmune and inflammatory diseases.

Previously published studies have demonstrated that using the CD58 fusion protein Alefacept to disrupt the CD2-CD58 interaction can inhibit T cell activation (51). More importantly, it was found that alefacept could specifically eliminate effector memory T cells in the peripheral blood and attenuate clinical symptoms in type-I diabetes and psoriasis (52, 53). Although the constructed peptides have biological activities *in vitro* and *in vivo*, their stability *in vivo* has limitations as most other peptides (54). Sable et al. adopted a novel approach to reinforce its stability *via* integrating the CD2 adhesion domain sequence from peptide 6 into the framework of rhesus theta defensins and sunflower trypsin inhibitor (55). The constructed cyclic peptides exhibit potent resistance toward enzymatic degradation and thermal denaturation. Among them, SFTI-a possesses a strong inhibitory activity of cell adhesion in the low nanomolar range to repress T cell-mediated immune responses from humanized arthritic mice (55).

## Soluble CD58

It was first discovered in 1993 by Hoffmann et al. that the presence of a soluble form of CD58 in human serum, urine, and cell supernatant *in vitro* (29). At high concentrations, sCD58 can bind to CD2-positive cells and restrain rosette formation of human T cells with sheep and human erythrocytes (29). The mixed lymphocyte reaction could also be profoundly dampened by sCD58 (46). Therefore, local release of large amounts of native sCD58 may disturb cell-cell adhesion and recognition *in vivo*. Besides, similar to suppression by CD58 mAbs, sCD58 alleviates the cytotoxicity of human NK clones (CD2^+^ CD3^−^). In contrast, sCD58 and mitotic CD2R mAb act synergistically in the triggering of T cell activation (46). These findings reveal that sCD58 modulates intercellular adhesion and T/NK cell-mediated immune responses by acting as a biological immunoregulator.

It has been shown that sCD58 can curb the lysis of neoplastic cells through competitively suppressing the binding to CD2. The release of substantial sCD58 from melanoma cells results in their accumulation within the tumor tissue at high concentrations sufficient to inhibit cellular immune responses and immunotherapeutic sensitivity (56). Hollander et al. found sCD58 was constitutively secreted into the supernatant of human B lymphoblastoid cells and the GPI-deficient mutant cells generated more sCD58 than wild-type cells (57). A similar phenomenon can be observed in lymphocytes from patients with paroxysmal nocturnal hemoglobinuria (PNH), which is characterized by a defect in the GPI-anchoring pathway. Therefore, lymphocytes in PNH patients generate more sCD58 than normal cells as the absence of GPI anchoring (58).

Although alternative splicing, direct secretion, and proteolytic shedding have not yet been corroborated as possible mechanisms of sCD58 production, the sCD58 release is likely to be derived from enzymatic cleavage of membrane-anchored CD58, since lack of a distinct mRNA for sCD58 and the downregulation of CD58 surface expression is always accompanied by the accumulation of sCD58 in the cellular supernatant (58–60). Furthermore, subsequent studies have revealed that the expression of surface CD58 is decreased following the treatment of PI-specific phospholipase C (PI-PLC) (61). Those changes between membranous CD58 and sCD58 may significantly affect adhesion/deadhesion processes, because the CD2-CD58 axis is one of the dominant pathways to mediate the interaction between T/NK cells and other cells (62, 63). Thus, cleavage of membranous CD58 may be responsible for the production of its soluble form, which plays a crucial role in the deadhesion of T/NK cells with target cells.

Regarding the immune function of sCD58, it was found that the dimeric and multimeric forms of synthetic sCD58 have a stronger potency than the monomeric biological form. The dimeric sCD58 inhibits antigen-stimulated proliferation of T lymphocytes to exert its immunosuppressive capacities *via* inducing regulatory T cells (64). The multimeric sCD58 is more effective than the monomer in refraining the proliferation of T lymphocytes in response to allogeneic cells, tetanus toxoid, or purified protein derivative (65). This inhibitory effect is not only due to physical blockage of intercellular interactions, but may also involve negative signaling generated *via* multimeric sCD58-CD2 interactions (65). Accordingly, it owns a strong potential as an immunomodulatory agent to suppress antigen-specific T cell responses for the treatment of inflammatory and autoimmune diseases. So far, the role of sCD58 in the tumor microenvironment has not been explored. With regard to the potential competitive inhibition of sCD58 between T/NK cells and target cells, sCD58 may be involved in cancer cell-induced immunosuppression, which has potential clinical implications and needs to be further mined and demonstrated.

## Effects of Cytokines and Drugs on CD58 Expression

The regulation of CD58 expression by cytokines is cell-dependent. In colonic epithelial cells, breast cancer cells and normal hepatocytic cells, the expression of CD58 is unresponsive to cytokine stimulation, including TNF-α, IFN-γ, IL-1, and IL-6 (66–68). There was no change in CD58 expression after stimulation of bronchial epithelial cells with TNF-α or IFN-γ (69). Similarly, TNF-α and IFN-γ do not influence the expression of CD58 in embryonic brain astrocytes (70). In contrast, the expression of CD58 was sensitively increased after incubation with IL-4 in human B-lymphoma cells and Burkitt’s lymphoma cell lines (68, 71, 72). Stimulation of cultured leukemic blasts with TNF-α increases CD58 expression, in turn facilitating susceptibility to lymphocyte-mediated lysis (73). After exposure to GM-CSF, CD58 expression is significantly upregulated in acute myelogenous leukemia (AML) cells (74). Besides, ultraviolet (UV)-B irradiation decreases the expression of CD58 on Epstein-Barr virus (EBV)-transformed B cells (75).

Notably, CD58 expression is significantly affected by some exogenous stimuli or drugs. The expression of CD58 on the surface of hepatocellular carcinoma (HCC) cells is dramatically elevated after anisomycin treatment and blockade of CD58 can potently impair the anisomycin-mediated enhancement of NK cytotoxicity (76). Thus, the adhesion molecule CD58 is likely to be critical for NK-mediated immunotherapy (76). Furthermore, β-interferon can significantly enhance the proportion of CD58 positive endothelial cells (77). All-trans retinoic acid (ATRA) and dexamethasone robustly diminish the surface expression of CD58 *in vitro*, which probably explains the efficacy of these drugs in treating inflammation-related diseases *in vivo* to some extent (78, 79). Moreover, long-term lead exposure reduces the expression of the erythrocyte adhesion molecule CD58, weakening the sensitivity to IFN-γ, in preschool children (80).

The surface CD58 appears to be unresponsive to cytokines, but the production of sCD58 is relatively sensitive to cytokines such as IL-1β, IFN-γ, and TNF-α. Albeit this, the generation of sCD58 varies from cell to cell, as demonstrated by its release from some, but not all, tumor cell lines. The sCD58 is only released in 6 out of 10 melanoma cell lines. Among them, sCD58 production can be potently affected by IFN-γ in all lines and by TNF-α in one (56). The sCD58 in the adenocarcinoma cell supernatant can be detected only after IL-1β stimulation (29). Both PMA and TNF-α can augment the release of sCD58 in HCC cells, but the production of sCD58 is unaffected following IL-1β stimulation (29). Thus, different cells exhibit different susceptibility to TNF-α and IFN-γ (29, 56). This regulation is cell-specific, especially IFN-γ, which inhibits the release of sCD58 in larynx epidermoid carcinoma cells but promotes the production of the soluble form in lung epidermoid carcinoma cells (60). In fact, CD58 is also present in a cytoplasmic “pool” of each cell; meanwhile, cleavage of surface CD58 by PLC can result in an increase of intracellular CD58 (60). Therefore, the cytoplasmic, membranous, and soluble form of CD58 is likely to be interrelated and dynamic. Apart from the expression level of CD58, activation status, secretory activity, and endogenous protein sheddase levels may be the major reasons for the cell-dependent differences in the production of sCD58 (60).

## CD2/CD58/CD48/CD59

CD2, CD48, CD58, and CD59 are tightly associated members of the immunoglobulin superfamily and they have similar structures in extracellular regions (81). CD58 is the primary natural ligand for human CD2; CD48 and CD59 are two additional, low-affinity ligands for human CD2, and their interactions in the human are limited and independent of glycosylation (82, 83).

The CD2 binding sites with CD58 and CD59 are overlapping, but not exactly identical (84). In murine T cell hybridomas expressing human CD2, anti-CD59 mAbs suppress CD2-mediated T cell activation, indicating that direct interaction of CD2 with CD59 likewise facilitates T cell-specific immune responses (84). Thus, CD59 is considered as the second ligand for CD2 and synergizes with CD58 to promote the adhesion and activation of T lymphocytes (85, 86). Notably, CD59 promotes CD58-mediated T cell proliferation and IL-2 production, whereas in the absence of CD2-CD58 interaction, the CD59 molecule itself cannot stimulate T cell proliferation alone even in the presence of exogenous recombinant cytokines such as IL-1, IL-6 (82).

**Figure 3:**
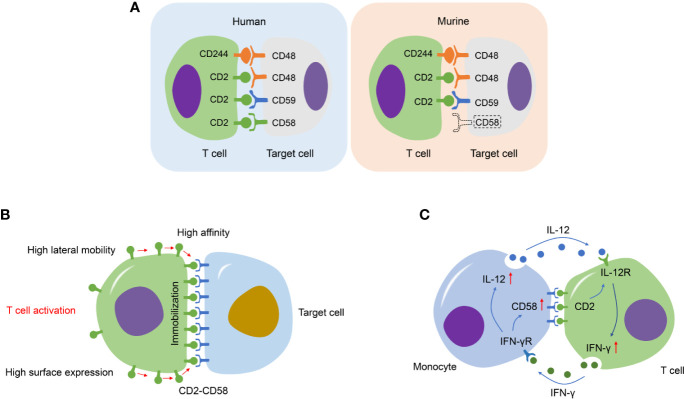
Schematic diagram regarding CD2/CD58/CD48/CD59 system, CD2-CD58 immobilization in T cell activation, and IL-12/IFN-γ feedback loop. **(A)** Specific differences of reciprocal adhesion molecule between human and murine in the CD2/CD58/CD48/CD59 system. CD2 and CD244 (2B4) are presented at the surface of T/NK cells in human and murine. Specifically, CD2-CD58 is the principal ligand-receptor pair. CD48 and CD59 are two additional and low-affinity ligands of CD2 in human. The CD48 receptor binds both CD2 and CD244, while the CD58 gene is absent in murine. **(B)** The immobilization of CD2-CD58 following T cell activation. This process consists of three important features, increased CD2 surface expression, the high affinity of CD2-CD58, and rapidly lateral mobility; a series of conformational changes is beneficial to strengthen intercellular adhesion and aid recognition. **(C)** The important role of CD2-CD58 interaction in the IL-12/IFN-γ positive feedback loop between monocytes and activated T cells.

Although CD58 is distributed on a wide range of human cells and tissues, the CD58 gene has not yet been found in murine, and the only counter-receptor for CD2 identified heretofore is CD48 (87). CD48 is considered to be a homologue of human CD58 in murine since its high similarities in distribution and structure (88). Arulanandam et al. surmise that CD58 may have evolved at the later stage of mammalian evolution due to gene duplication from CD48 to become an exclusive counter-receptor for CD2 after divergence from murine (89–91). The species-specific differences in the CD2/CD58/CD48/CD59 system are summarized in **Figure 3A** (91).

In humans, T/NK cell adhesion molecule CD2 interacts with diverse ligands, such as CD58, CD48, CD59, and even the novel carbohydrate structure (92). However, there is no additional ligand for the adhesion pair of CD2-CD48 in murine (93). The interaction affinity of mouse CD2-CD48 is lower than that of human CD2-CD58. Murine CD48 is also involved in the modulation of T cell activation, and CD48 binds to the T11 (1) region of CD2, the identical area of CD2 interacts with CD58 (94). Application of anti-CD48 mAb can effectively restrain not only weak, hapten-specific responses, but also strong, alloantigen-specific responses of cytotoxic T lymphocytes (CTLs) *in vivo* (95). Besides, anti-CD48 mAb interferes with CD4^+^-dependent pathways *in vivo*, and the maximal effect of it concentrates on the immune efferent stage (95). Of note, combined administration of CD48 with CD2 mAbs cannot heighten the immunosuppressive effect generated by CD2 mAb alone, indicating that regulation of the CD2 receptor, rather than a disturbance of the CD2-CD48 interaction, is the primary effect of CD2-mediated immunosuppression in the murine (96).

## Immunological Synapse

After successful recognition of APCs by T cells, a specialized nanoscale structure is formed in the contact area through cytoskeletal remodeling and receptor rearrangement, known as the IS (97). Specifically, the IS consists of three layers of supramolecular activation complexes (SMAC), classified into central, peripheral, and distal SMAC (c, p and dSMAC, respectively) (98). The CD2-CD58 interactions are important components of the IS and contribute to the maintenance of high intracellular calcium levels (99). In addition to localizing to cSMAC together with other molecules, including CD28/CD80/86 and TCR-peptide-MHC complexes, the clusters of CD2-CD58 complexes also shape a ring-like framework between pSMAC and dSMAC at the outer edge of the mature IS, termed “corolla” (**Figure 1C**) (100). The corolla amplifies the activity of p-SFK/LAT/PLC-γ superior to TCR alone. The CD2-CD58 interactions in corollas are more signal-enhancing than central CD2-CD58 interactions. The corolla boosts CD2-dependent amplification of TCR signaling but can be buffered by PD-1 invaded the corolla (101). Chimeric antigen receptor (CAR) T-cell transfer is a novel and promising approach of adoptive T-cell immunotherapy in tumors. When in contact with the target cancer cell, CAR-T cell form an important IS with cancer cell, in turn dynamically coordinating multifarious forces to execute its cytotoxic function (102). Strategies to assist CAR-T-mediated IS with tumor cells by strengthening CD2-CD58 interaction may be beneficial for cancer immunotherapy.

## CD2-CD58 in T Cells

T cell activation results in increased CD2 surface expression, affinity, and lateral mobility, allowing the CD2 molecules to diffuse from the lateral area into the contact interface and engage CD58 (103, 104). Afterwards, this process causes the CD2 that binds CD58 to be recognized and immobilized at the region of intercellular contact *via* CD2 conformational change, thus elevating the number of CD2-CD58 ligations and strengthening adhesion (103). This pattern combines passive diffusion with active recognition of conformational alteration to potentiate intercellular adhesion by CD2-CD58 interactions (**Figure 3B**).

In the absence of costimulatory signaling, stimulation of T cells by TCR/CD3 alone results in T cell anergy (105), thus the activation of T lymphocytes requires at least two signals. The first signal provided by ligation of the TCR with a specific MHC, and the second signal involves the ligand-receptor pair interactions of costimulatory adhesion (106–108). CD2 is one of the most important adhesive receptors presented on almost all of T lymphocytes and it offers costimulatory signal after interacting with CD58 on target cells (12, 109). The purified CD58 possesses a clearly mitogenic effect for human resting T lymphocytes (110). The anti-CD2 and anti-CD58 mAbs induce T cell unresponsiveness to mitogenic or antigenic stimuli and inhibit CTL-mediated killing by binding to the T cells and target cells, respectively (111). These results reveal the critical role of the CD2-CD58 interaction in T cell stimulation.

Although the CD2 engagement by CD58 alone is not sufficient for T cell activation (111–113), the CD2-CD58 interaction without other stimuli can still trigger intracellular biochemical alterations, that is, modulation of T cell function by inducing remarkable, transient upregulation of intracellular cAMP concentration (114). In the absence of TCR stimulation, CD58-bound CD2 induces signaling into microdomains *via* the actin-dependent aggregation of signaling molecules, such as LAT, Lck, and TCR-ζ chain (115). When stimulated together, TCR and CD2 were separated to different regions after transient colocalization in small microdomains; this spatial segregation is likely to allow the two receptors to synergistically strengthen signal transduction (115). Both receptors with different structures induce a fast spatial reconstruction of molecules in the cell membrane, indicating a pattern that local accumulation of signaling molecules initiates T cell signaling (115). Moreover, CD2-CD58 interaction renders the generation of a close adhesion zone between T cell and APC, in which the binding of TCR to peptide-MHC complexes is potentiated (116). TCR drives PLCγ1 phosphorylation and increases the enzymatic activity of PLCγ1, resulting in phosphoinositide cleavage and continuous Ca2^+^ mobilization, which is necessary for T cell proliferation and cytokine production (117, 118). The CD2-CD58 interaction is able to maintain and reinforce antigen-mediated Ca2^+^ influx in T lymphocytes interacting with APCs. CD2 and TCR is synergistic, and their signals converge to activate the PLCγ1/Ca2^+^ pathway at the IS (99).

The costimulatory signaling of CD58 activates CTLs to proliferation, cytotoxicity, and cytokine secretion, including IFN-γ, TNF, and IL-2 (119). IL-2 is the main T cell growth factor transcribed in resting T lymphocytes (120). As an important secondary signal of T cell activation in response to CD58-positive antigen-bearing stimulator cells, CD2-CD58 signaling induces IL-2 secretion through influencing nuclear factor (NF)-mediated the transcription of the IL-2 promoter-enhancer (121, 122), which maintains autocrine T cell growth and the generation of IFN and TNF (123). Furthermore, in the presence of CD58-like signals, such as human rCD58, T cell responsiveness to both IL-6 and IL-1 is promoted by CD2-CD58 interaction, suggesting it exerts a significant function in T cell/monocyte interactions during the initial immune responses *via* increasing T cell sensitivity to monocyte-secreted cytokines (124). Costimulation of T lymphocytes by CD58 effectively facilitates IFN-γ and IL-10 secretion in a calcineurin-dependent manner, and both IFN-α and IL-12 can further increase CD58-mediated IL-10 secretion (125). In contrast, TNF-α, IL-2, IL-4, IL-5, IL-13 production is low or even absent following CD58 costimulation, which was not an inhibitory effect of endogenously produced IL-10 (125). Furthermore, T regulatory cells (Tregs) are relatively poor in terms of mediation of Th1/Th2 immune responses, secretion of IL-10, and proliferation responses *in vivo* (126). CD2-CD58 interaction can induce the of non-proliferative Tregs with the production of large quantities of IL-10. This effect is unique to CD2 signaling since it is not acquired or even suppressed *via* mobilizing other costimulatory (127).

Of note, the CD2-CD58 interaction can particularly improve the T lymphocyte response to IL-12, which possesses a series of immunoregulatory effects on activated T/NK cells, like proliferation stimulation, IFN-γ secretion, and cytotoxicity (128). IL-12 responsiveness to APC-depleted T lymphocytes is restored by the Chinese hamster ovary (CHO) cells expressing CD58 (129). More importantly, the CD2-CD58 interaction offers the central functional connection in the IL-12/IFN-γ positive feedback loop between monocytes and activated T cells (**Figure 3C**) (130). During antigen presentation, a sufficient number of CD58 molecules on monocytes bind to the amino-terminal domain of CD2 on T cells. Relating intracellular signals by CD2 subsequently generates and initiates optimal T cell responsiveness to IL-12 (131). Monocyte-secreted IL-12 induces Th1 differentiation and significantly increases cytokine secretion, including IL-2 and IFN-γ (129). In turn, T cell-derived IFN-γ motivates monocytes to produce IL-12 and boosts the expression of CD58 in monocytes, thus further strengthening CD2-mediated signaling and maintaining T cell responsiveness to IL-12 (131). Moreover, IFN-γ provokes monocyte to kill the intracellular pathogen, whereas IL-12 and IL-2 facilitate non-MHC-restricted NK cell killing. Therefore, the CD2-CD58 interaction may be regarded as an important part of innate and acquired immune responses.

One of the most important factors causing activation-induced cell death (AICD) of T cells, an essential sustainer for lymphoid homeostasis, is triggered by the ligation of Fas (Fas-L) (132). Fas-induced AICD of activated T cells is effectively protected by dendritic cells (DC) in a CD58-dependent fashion (133). More importantly, CD2-CD58 interaction potently refrains the apoptosis of T cells through blocking the CD3-mediated Fas/Fas-L upregulation (134). CD58 costimulation increases the number of effective nuclear NF-ATp and maximizes the induction of NF-AT complexes, implying CD2-CD58 signaling is implicated in the regulation of NF-AT translocation from cytosol to nucleus (122). In addition, costimulation of CD2-CD58 on primary T cells results in STAT1 phosphorylation and nuclear translocation (135). Notably, cytokine-driven STAT phosphorylation is usually transient, whereas STAT1 phosphorylation upon CD2-CD58 stimulation can sustain several days. Transcription of pivotal target genes, including c-fos and IRF1, undergoes prolonged and delayed effects after CD2 stimulation, hinting that the special model of STAT activation may incur a unique cellular response following CD2 stimulation by CD58. Interestingly, this signaling seems to be exclusive to T cells, CD2 stimulation on NK cells cannot evoke STAT1 phosphorylation (135).

A small fraction of human CD3^+^ T cells are known to co-express CD56 (136), an antigen generally restricted to NK cell expression. It has been demonstrated that CD3^+^ CD56^+^ T cells have strong MHC-unrestricted cytotoxicity against neoplastic cells *in vitro* and *in vivo* (137). The CD2-CD58 interaction precisely provides the strong activation signals for expansion and differentiation of CD3^+^ CD56^+^ T cells (138). In adults, a considerable proportion of CD8^+^ T lymphocytes lack the expression of CD28, which is one of the characteristics of T cell senescence, meaning a low proliferative capacity and functional impairment (139). The majority of costimulators have a low ability to activate CD28-deficient T cells, while the CD2-CD58 interaction strongly induces the proliferation and cytokine production, as well as enlarges TCR signals in CD28^−^ CD8^+^ T cells (140). Blocking CD58 significantly dampens the response of CD28^−^ CD8^+^ T cells to allogeneic DCs and viral antigens (140). These results reveal that CD2-CD58 signal is an important costimulatory pathway to facilitate the control of chronic infection by maintaining the persistent expansion of CD28^−^ CD8^+^ T cells.

Apart from T/NK-mediated cellular immunity, it is worth noting that CD2-CD58 interaction also participates in the immunoregulation of humoral immunity. Recently, the CD2 and CD58 homologs in the model species zebrafish have been identified, which have the same conserved structural characteristics as mammals (141). After antigen stimulation, CD2 and CD58 on CD4^+^ T cells and APCs are increased, respectively. Loss function of CD2 and CD58 strikingly restrains the activation of mIgM^+^ B cells and antigen-specific CD4^+^ T cells, and subsequently suppresses the production of antibody and host defense against pathogens. The CD2-CD58 interaction offers a major costimulatory signal for the sufficient activation of CD4^+^ Th-mediated adaptive humoral immunity in zebrafish (141). Given the absence of CD58 in rodents, zebrafish is anticipated to serve as an animal model for immunological research to make up for the shortcomings of mouse models.

## CD58 in Thymocyte Development

During the differentiation and development of thymocytes, CD2 is one of the earliest molecules expressed; its surface density gradually reduces as thymic maturation (7). The CD2-CD58 interactions influence the affinity between TCR and peptide-MHC at the stage of positive and negative selection, which confers the ability of immature thymocytes to resist the high affinity of TCR-pMHCs to escape negative selection (100). Thymocyte proliferation needs the induction of CD58-positive L cells and phytohemagglutinin (PHA), which could be repressed by CD2 or CD58 mAb (142). Receptors for CD2 antigens situated on reticular epithelial cells, which can initiate the induction of proliferative wave of immature cortical thymocytes through interacting with the CD58 molecule (143). The anti-CD2 and anti-CD58 mAbs impede the binding of thymocyte with thymic epithelial cells, and thus suppress thymocyte activation in thymic epithelial cell-dependent manner, meaning that the natural ligand CD58 presented on human thymic epithelial cells contributes to the T cell mature and activation *via* the CD2 molecule (62, 144). Collectively, these results outline a crucial role for the CD2-CD58 pathway in T cell maturation and thymic differentiation.

## CD58 in NK Cells

At a study of the mechanism that NK-mediated cytotoxicity to breast cancer targets, unexpectedly, anti-CD58 mAb failed to inhibit NK-mediated killing but instead mediated the enhanced cytotoxicity associated with CD58 expression, albeit CD2 blockade mildly reduced cytotoxicity (145). These results indicate NK-mediated cell lysis of breast cancer is potentiated through antibody-dependent cellular cytotoxicity (ADCC) against CD58. More importantly, CD2-CD58 interaction exerts an important function in cytotoxic function and membrane nanotube formation between NK cells and target cells (146), which is a wafery membranous protrusion physically linked two cells and able to perform substantial functions including assisting in cell-to-cell communication (147). It reveals a special role for CD2-CD58 in allowing NK cells to explore the local microenvironment through facilitating nanotube formation.

Notably, CD58 is also expressed in NK cells. Freshly isolated NK cells from human peripheral blood are consistently CD58-positive and activated NK cells with IL-2 *in vitro* results in an approximately 5-fold increase in surface expression of CD58 (148, 149). Therefore, CD58 appears to exert dual or even multiple functions. However, the exact function of CD58 on NK cells to date is still unclear. Future research should focus on this issue and investigate the functional differences of CD58 in immune cells and target cells, which is critical for therapeutic applications.

## CD58 in Other Immune Cells

The surface of memory T cells express high levels of CD58, which has an important role in improving their responsiveness, and the CD58^+^ subgroup generates more IFN-γ than the CD58^−^ subgroup following PHA stimulation (150). In terms of DCs, the significant role of the CD2-CD58 interaction in DCs is to enable immune and non-immune cells to directly interact with DCs, triggering innate and adaptive immune responses (151). Besides, CD2-CD58 interaction has been reported to participate in B cell differentiation by interacting with T cells and monocytes to some extent, but not in its proliferation (152). The binding of CD2 with CD58 located on the surface of autologous erythrocytes increases B cell responses to mitogens and antigens (153). Antibodies against CD58 can induce IgE secretion in IL-4-activated B cells (154). Thus, CD2-CD58 stimulation provides alternative signaling to modulate IgE production through intercellular contact interaction.

## CD58 in Endothelial Cells

CD58 molecule plays a critical role in the interactions between T cells and ECs. Early costimulation by EC facilitates lipid raft clustering in a CD2-CD58 dependent manner, resulting in the enhancement of TCR-triggered pathways (155). Human ECs increase the expression level of CD40 ligand, a vital receptor mediating T cell activation, in activated CD4^+^ T cells *via* CD58-induced mRNA stabilization (156, 157). Furthermore, CD58 can fuel T cell adhesion to EC, facilitating the recruitment of circulating T lymphocytes into the inflammation site *in vivo* (158). The blockade of CD58 dampens T cell-mediated cytotoxicity to allogeneic EC and impairs IL-2 transcription and cytokine synthesis of EC (159–161). Activated T cells can enhance the permeability of ECs by the CD2-CD58 interaction (162).

## CD58 in Intestinal Epithelial Cells

It has been found that CD58 is expressed constitutively in the native IEC and IEC lines. Anti-CD58 mAb suppresses IEC-mediated proliferation of CD4^+^ T cells (163). Specifically, CD58 molecules are highly polarized and confined to the basolateral surface of the IECs in a topological fashion at the contact area of T cells, and act as a costimulator in HLA class II-mediated antigen presentation (163). Moreover, intestinal CD3^+^ TCRαβ^+^ CD8^+^ intraepithelial lymphocytes (IEL) are strongly linked to IECs and CD2-CD58 interaction participates in their crosstalking. Concretely, IELs are stimulated *via* interacting with IECs by the CD2-CD58 pathway and this process promotes the synergistic synthesis of IL-8, leading to the TNF-α release, which in turn increases IL-8 production and CD58 expression by the IECs (164).

## CD58 Polymorphisms

It has reported that CD58 single-nucleotide polymorphisms (SNP), including 6 variations, rs12044852A/C (SNP1), rs2300747A/G (SNP2), rs1335532C/T (SNP3), rs1016140G/T (SNP4), rs1414275C/T (SNP5) and rs11588376C/T (SNP6), related to the risk of neuromyelitis optica (NMO) (165). For instance, rs1016140 G allele can enhance T cell activity and impede the penetration of AQP4 antibody into the central nervous system (CNS), eventually causing NMO progression. The rs2300747 A allele augments NMO risk by reducing the RNA expression of CD58. Furthermore, the percentage of CD58-positive monocytes is markedly lower in healthy controls with each of these risk genotypes of autoimmune thyroid diseases (AITDs), and lower in patients with Graves’ disease and Hashimoto’s disease, compared to healthy individuals (166). Therefore, CD58 SNPs may participate in AITD susceptibility by decreasing CD58 expression. In a large cohort of candidemia, Kumar et al. analyzed more than 110,000 SNPs at 186 loci known to date to be related to immune-mediated diseases and showed a strong correlation between CD58 SNPs and candidemia (167). Altered level of CD58 not only modulates macrophage phagocytosis, but also indirectly affects cytokine production. For example, the SNP rs17035850 of CD58 is relevant in persistent fungemia, a positive blood culture lasted for 45 days albeit sufficient treatment, whereas the SNP rs12025416 of CD58 is linked to lower levels of Candida stimulated TNF-α and IL-6 (167).

## Multiple Sclerosis

MS is a genetically complicated autoimmune disease in the CNS. Many published studies have illustrated that CD58 SNPs such as rs12044852 and rs2300747 are tightly related to MS risk in different populations, including European Caucasian, Iranian, Russians (168–172). A recent study found that carriers of the MS risk allele rs1414273 exhibited decreased CD58 mRNA levels but elevated miR-548ac levels through analyzing diverse datasets from global populations (173). In particular, SNP rs1414273 is localized at the miR-548ac stem-loop site of CD58 first intron, which can regulate the cleavage activity of Drosha, thus propelling expression uncoupling between CD58 and miR-548ac from a common original transcript in immune cells (173). Additional evidence for the role of CD58 in MS susceptibility reveals that CD58 expression is reduced in the cerebrospinal fluid of patients with MS (174). Genome-wide association scans demonstrated that CD58 allelic variants were linked to the risk of developing MS *via* analyzing more than five thousand MS patients (170). The CD58 protective allele (rs2300747) of MS exerts its function on disease risk through elevating CD58 mRNA expression in a dose-dependent fashion in circulating mononuclear cells and lymphoblastic cells from MS patients during clinical remission (170). Mechanistically, protective allele-induced CD58 accumulation increases the expression of the transcription factor FoxP3 *via* CD2-CD58 interaction, potentiating the function of CD4^+^ CD25^high^ Tregs that are defective in MS (170). Moreover, the protective allele of rs1335532 is associated with MS and is located in the active enhancer region of the CD58 gene, generating a strong functional binding site of Ascl2, which induces activation of the CD58 promoter *via* the Wnt pathway in monocytes and lymphoblasts (175). Notably, the Alu insertion facilitates skipping of CD58 exon 3 and drives a frameshifted transcript, suggesting that Alu polymorphism is perhaps a causative factor for elevated MS risk (176).

## Chronic Hepatitis

The expression of CD58 in hepatocytes of chronic hepatitis exhibits cytoplasmic and membranous staining and elevated with the severity of chronic HBV infection, the degree of inflammatory activity, and liver damage (177–179). More importantly, the proportion of CD58^+^ cells in peripheral blood mononuclear cells and the levels of sCD58 in serum of patients with HBV infection are conspicuously higher than that in the healthy individuals and positively associated with serum levels of AST and ALT (178, 179). These findings demonstrate that CD2-CD58 interactions between lymphocytes and hepatocytes exert an essential function in chronic hepatitis (177). Immune adhesion molecule CD58 may strengthen viral elimination *via* activating T/NK cells and stimulating the cytotoxic immune response. Unfortunately, this also causes the damage of hepatocytes (179).

## Rheumatoid Arthritis

The level of CD58 in chondrocytes is higher in arthritic joints than in normal joints; CD58 expression is higher on synovial fluid lymphocytes of RA in comparison with peripheral blood lymphocytes from RA patients or healthy individuals (180). The expression of sCD58 in synovial fluids and serum from patients with RA are remarkably diminished in contrast with that in control subjects and patients with spondyloarthropathy (SpA) or osteoarthritis (OA) (180). Under physiological conditions, the CD2-CD58 interaction could be inhibited by local sCD58 production. Therefore, the insufficient release of sCD58 may lead to accumulation of T cells and continued inflammation in synovitis due to sCD58-mediated deadhesion (181).

## Cytomegalovirus Infection

CMV is the main pathogen in AIDS patients and transplant recipients, and the presence of this virus can exacerbate allograft rejection. The surface expression of CD58 augmented after CMV infection *in vitro*, caused by direct action of virus infection rather than by a secondary induction of cytokine (182). The CD2 interaction with increased CD58 on the surface of CMV-infected cells is a crucial node for antibody-induced activation and NK-mediated cytotoxicity during the antiviral response (183). Blockade of CD2-CD58 interaction causes a reduction in the secretion of TNF-α and IFN-γ by adaptive NK cells following CMV infection. As a virus-encoded downregulation factor of CD58, the CMV glycoprotein UL148 can retain CD58 within the endoplasmic reticulum without being transported to the cell surface, which weakens activation of CTLs and attenuates cell-mediated antiviral response (184). Therefore, CD2-CD58 interaction is critical for the recognition and activation between T/NK cells and CMV-infected cells.

## Inflammatory Bowel Disease

Serum levels of sCD58 are profoundly reduced in IBD, including Crohn’s disease and ulcerative colitis, relative to healthy controls. Decrease of sCD58 in sera associated with multiple clinical parameters of disease activity, including CDAI score and erythrocyte sedimentation rate (ESR) (185).

## Transplantation

Co-expression of CD58 on the stimulator cells elicits significant potentiation of the primary alloresponse and proliferative response of CD4^+^ T cells (186). In the rat model of heart transplantation, treatment with CD2-targeting mAbs conspicuously prolong rat survival (187). Although anti-CD48 mAb alone fails to prolong graft survival, anti-CD48 mAb can synergize with anti-CD2 mAb to induce long-term survival of allograft (187, 188). Another xenograft mouse experiment shows that blocking the CD2-CD58 axis effectively prevents human skin allografts from lymphocyte infiltration and inflammation damage (189). Therefore, the CD58 molecule plays a role in lymphocyte-mediated immune rejection, and blockage of CD2-CD58 interaction contributes to alleviating allograft and xenograft responses.

## Hematological Malignancies

### Acute Lymphoid Leukemia

In ALL, CD58 expression is negatively related to the percent of peripheral blast cells, leukocytosis, and the presence of a clinical tumoral syndrome (190). Leukemia patients with poor prognosis frequently lack the expression of CD58, while the higher expression of CD58 is strongly associated with longer survival time (191). In addition, CD38^+^ CD58- is an independent poor prognostic factor in pediatric patients with Ph^−^ B-cell ALL, who have shorter survival and higher risk of relapse (192). As nonmalignant B cells differentiate from early to mature stages in the bone marrow, the expression of CD58 gradually reduces, while it is usually upregulated in pediatric and adult B-cell ALL (193). The expression of CD58 is remarkably higher in ALL blasts than that in normal B cells, whereas there is no significant difference between regenerated and normal B cells (194). More importantly, CD58 has high accuracy and stability in minimum residual disease (MRD) detection at different clinical stages, thus CD58 could be used as an effective indicator for monitoring MRD in B-cell progenitor ALL (BCP-ALL) (194, 195). Furthermore, due to the presence of hematogones, it may be difficult to distinguish leukemic lymphoblasts in the diagnosis and follow-up of BCP-ALL. The use of the CD81/CD58 ratio as the discriminating marker enhances the difference between leukemia lymphoblasts and hematogones with high sensitivity and specificity in patients with BCP-ALL (196).

### Acute/chronic Myelocytic Leukemia

In AML, CD58 expression is positively correlated with complete remission rate, overall survival, and disease-free survival (191). Progenitor cells from untreated CML patients exhibit diminished CD58 expression, but surface CD58 expression could be at normal levels or even exceed normal levels after IFN-α treatment (197). CML progenitor cells lacking CD58 cannot activate normal proliferation responses of T lymphocytes, resulting in abnormal adhesion of CML progenitor cells and abnormal clonal proliferation (197). Transformed cells are generally killed by lymphokine-activated killing (LAK) cells. Anti-CD58 mAb can significantly block the LAK cell lysis, indicating the loss of CD58 in CML may be an important cause of LAK resistance (198).

## Lymphoid Malignancies

### Burkitt’s Lymphoma

The absence of CD58 expression is a common feature of BL, which helps tumor cells escape immunological surveillance (199). The BL cells form conjugates with EBV-specific CTLs *via* the LFA-1/CD45 pathway, but these conjugates fail to evoke target cell lysis in the absence of the CD2-CD58 interaction, suggesting the crucial effect of CD58 in activating EBV-specific CTLs (200). To some extent, the loss of CD58 in EBV-positive BL is the basis for neoplastic cells to evade virus-specific T cell control.

### Hodgkin’s Lymphoma

The formation of T cell rosettes in HL relied on the IS, and activation of rosetting T lymphocytes is dependent on the CD2-CD58 interaction (201). Although CD58 mutations in primary Reed/Sternberg (HRS) cells are rare, inactivating mutations in CD58 are common in HL cell lines and relapsed HL patients (202, 203). At the advanced stage of HL, CD58 inactivation of HRS cells located in pleural effusions is extremely prevalent, which provides favorable conditions for the immune escape of tumor cells (202).

### Diffuse Large B Cell Lymphoma

Recently, several studies have reported that CD58 plays a key role in the pathophysiology of DLBCL. Genomic inactivation or mutation of CD58 causes loss of surface expression that is an independent adverse prognostic factor in DLBCL (204). An attenuation in T/NK-mediated cell lysis in DLBCL can be restored by re-expression of wild-type CD58 (205), indicating the absence of CD58 is beneficial to disturb recognition between DLBCL cells and T/NK cells in a CD2/CD58-dependent manner to evade immunosurveillance. Besides, EZH2 inhibitor can restore CD58 expression on the surface of lymphoma cells, which in turn increases IFN-γ secretion of T/NK cells against lymphoma cells. Mechanistically, there is a highly trimethylated H3K27 in the promoter region of CD58, which induces CD58 gene silencing and mediates immune escape of lymphoma cells, whereas EZH2 inhibitor can effectively rescue epigenetic repression of CD58 expression through boosting its demethylation and activating CD58 gene transcription (206). In addition to DLBCL, the CD58 gene is also one of the recurrent targets of genetic abnormalities in other lymphoid malignancies, such as acute adult T cell lymphoma and peripheral T cell lymphoma (207, 208). Taken together, these studies support the notion that the CD58 molecule plays a vital role in tumor cell biology and highlight that regulation of the adhesion molecule CD58 on the surface of tumor cells may be a promising immunotherapeutic strategy.

### Solid Tumors

An increasing number of studies have revealed that the CD58 molecule plays the crucial roles in immune evasion of solid tumor cells. In neuroblastoma, CD58 is critical for the susceptibility of it to the cytotoxic effects of LAK and NK cells. Blocking CD58 on neuroblastoma cells could attenuate NK and LAK cytotoxicity (209). In colorectal cancer (CRC), Lorenz et al. (210) constructed a recombinant virus bearing CD58 (rv-CD58) to evaluate the role of CD58 on neoplastic cell immunogenicity. CRC cells infected by rv-CD58 were potently positive for CD58 and effectively potentiated intercellular adhesion, stimulated the T cell proliferation, and augmented CTL cytotoxicity. In the immunocompetent C57BL/6 mice model, rv-CD58-infected murine CRC cells significantly refrained tumor growth and induced antitumor immunity (210).

In addition to mediating T immune response in solid tumors, several recent reports have demonstrated that CD58 molecule can serve as stem cell marker or an oncogene in tumor initiation and progression. Xu et al. (211) found that CD58 was highly expressed in CRC, CD58-positive tumor cells were frequently present in primary specimens and CRC cell lines, and demonstrated increased tumorigenicity *in vitro* and *in vivo*. More importantly, elevated CD58 facilitated the self-renewal of CRC-initiating cells through activating the Wnt/β-catenin pathway by degradation of Dickkopf 3. Besides, CD58 silence notably dampened sphere formation and tumor growth (211). In gastric cancer (GC), high levels of CD58 are associated with cell dedifferentiation, invasion of tumor cells into lymph and blood vessels, decreased survival time, and cancer recurrence (212). Primary tumors and metastatic lymph nodes showed extensive expression of CD58. Furthermore, distant metastases, such as peritoneum and liver, have consistently high proportions of CD58^+^ GC cells (212), indicating CD58 provides a selective advantage for GC cells to establish novel distant metastatic sites. Notably, upregulation of CD58 expression in tumors appears to contradict its role as a T cell costimulatory molecule, as high expression of CD58 on the surface of tumor cells may be more readily recognized and killed by T cells. Therefore, the function of CD58 in tumor cells is not simple and isolated, but complex and diverse, and needs to be further investigated in depth.

Although immune checkpoint blockade (ICB) therapy has exhibited unprecedented clinical efficacy in tumor treatment (213, 214), ICB still lacks efficacy in the majority of cancer patients (215). A recent study reported that the surface expression of CD58 was strongly reduced in tumor cells of melanoma patients with ICB resistance compared with that of untreated patients (216). CD58 loss induced immune evasion in different co-culture models with CTLs, and the PD-L1 expression was elevated in CD58-knockout melanoma cells (216). These data illustrated that the loss of CD58 facilitated immune evasion possibly *via* different mechanisms, including deficiency of T cell costimulation, reduction of T cell adhesion, and even synergy of the corepressor PD-L1. Thus, elevating CD58 expression is likely to contribute to the alleviation of ICB resistance in melanoma patients. In particular, the expression of CD58 was independent of the IFN-γ pathway, and the loss of CD58 led to immune escape without affecting MHC expression (216), indicating that it differs from the known mechanisms of ICB resistance.

## Conclusion

**Figure 4:**
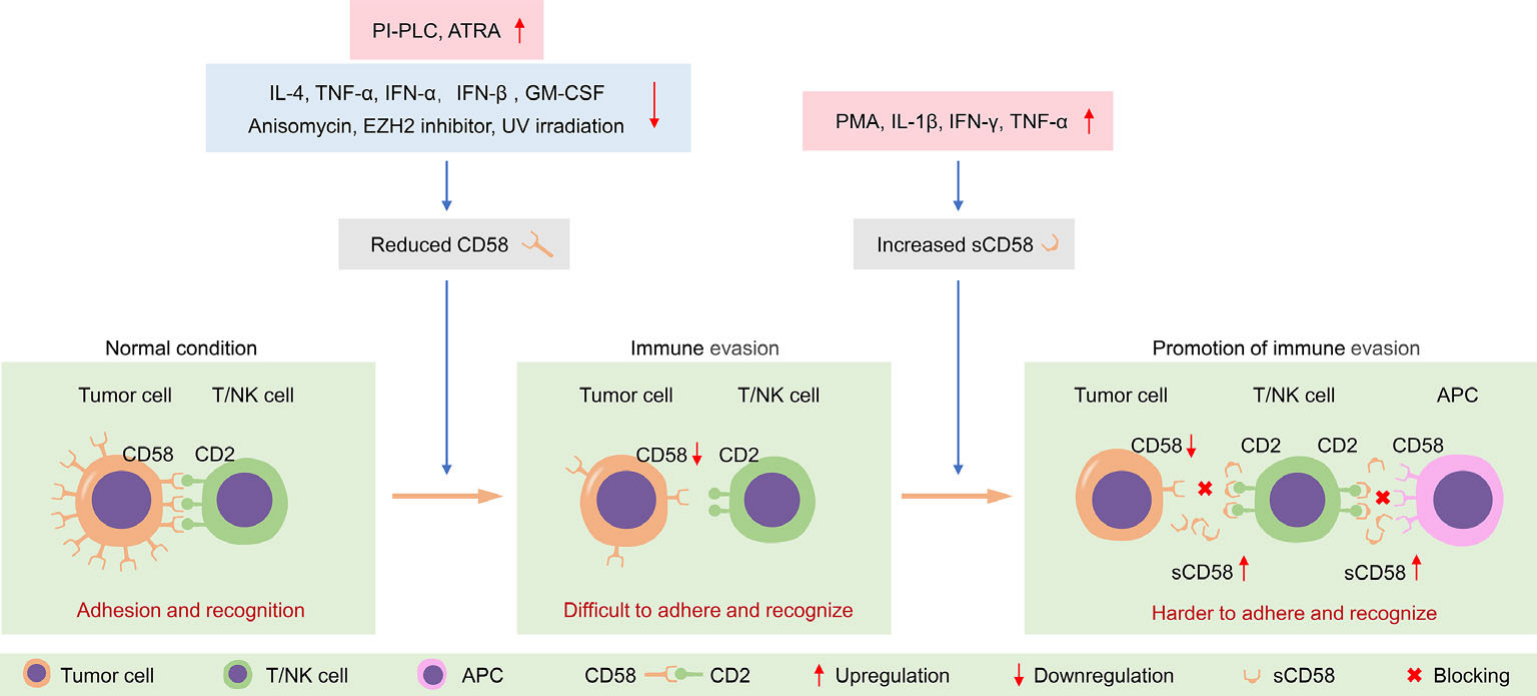
Tumor cells evade immune surveillance by regulating the expression of CD58 on the membrane surface and sCD58 in the microenvironment. PI-PLC, phosphatidylinositol-specific phospholipase C; ATRA, all-trans retinoic acid; UV, ultraviolet; PMA, phorbol-12-myristate-13-acetate; APC, antigen-presenting cell.

Herein, we have comprehensively summarized CD58 isoforms, sCD58, CD2-CD58 interaction, their structure and function, IS formation, CD58 polymorphisms, meanwhile discussed the crucial roles of CD58 as a costimulatory molecule for T/NK cell-mediated immune response in tumor-related and immune-related diseases. Regarding the roles of CD58 in tumor immunology, a looming but promising picture begins to come into sight from current studies (**Table 1**). On the one hand, loss of surface-related CD58 expression attenuates the susceptibility of tumor cells to CTL-mediated cytolysis; on the other hand, the local accumulation of sCD58 in the tumor microenvironment is likely to interfere with the adhesion and recognition of T/NK cells by serving as a natural immunosuppressor (**Figure 4**). The sCD58-mediated interference is not only for the recognition of the tumor cells themselves, but also includes T cell interaction with APCs. Only CD3/TCR-related signals alone without CD2-CD58 costimulatory signal may result in T cell anergy. Of note, the inhibitory effect incurred by sCD58 in the microenvironment is not only due to physical blockage of cell-cell interactions, but may also involve negative signaling inside T/NK cells through sCD58-CD2 interactions. These CD58-mediated possible mechanisms facilitate immune evasion and metastasis of tumor cells, although further in-depth studies are needed. The ideal clinical application model of CD58 in cancer immunology is to stimulate the surface expression of CD58 on cancer cells and to inhibit the secretion of sCD58 into the tumor microenvironment, however, there still remains several pending questions (1): the molecular mechanisms of sCD58 production (2); the roles of sCD58 in varieties of cancers (3); values as a therapeutic target in autoimmune diseases and malignant tumors.

**Table 1:** Expression, function and clinical significance of CD58 in various malignancies.

Malignancy types	Expression	Functions	Mechanisms	Clinical characteristics	Prognosis	References
Acute lymphoid leukemia	Downregulated	NA	NA	The percent of peripheral blast cells, leucocytosis, and the presence of a clinical tumoral syndrome	Overall survival	(190, 191)
B-cell progenitor ALL	Upregulated	NA	NA	Identification marker; the detection of minimum residual disease	NA	(194–196)
Acute myelocytic leukemia	Downregulated	Evading immunosurveillance	NA	Complete remission rate	Overall survival and disease-free survival	(191)
Chronic myelocytic leukemia	Downregulated	Abnormal adhesion of CML progenitor cells and abnormal clonal proliferation of T cells	NA	NA	NA	(197)
Burkitt’s Lymphoma	Downregulated	Evading immunosurveillance	NA	NA	NA	(200)
Hodgkin’s lymphoma	Downregulated	Immune evasion	NA	At the advanced stage of HL, CD58 inactivation of HRS cells located in pleural effusions is extremely prevalent	Relapse of HL	(202, 203)
Diffuse large B cell lymphoma	Downregulated	Evading immunosurveillance	Inhibiting IFN-γ secretion of T/NK cells against lymphoma cells	NA	An independent adverse prognostic factor	(204–206)
Neuroblastoma	NA	Susceptibility of it to the cytotoxic effects of LAK and NK cells	NA	NA	NA	(209)
Hepatocellular carcinoma	Upregulated in anisomycin-treated HCC cells	Promotion of immune synapse formation to boost NK-mediated immunotherapeutic effects	NA	NA	NA	(76)
Gastric cancer	NA	NA	NA	Cell dedifferentiation, invasion of lymph and blood vessels, distant metastases	Overall survival and disease-free survival	(212)
Colorectal cancer	NA	Potentiation of intercellular adhesion, stimulation of the T cell proliferation, and augment of CTL cytotoxicity	NA	NA	NA	(210)
	Upregulated	Enhancement of sphere formation, EMT ability and tumor growth; Promotion of self-renewal of cancer stem cell	Activating the Wnt/β-catenin pathway by degradation of Dickkopf 3	NA	NA	(211)
Melanoma	Downregulated in patients with ICB-resistance	Immune evasion	Deficiency of T cell costimulation, reduced T cell adhesion, and even synergy of the corepressor PD-L1.	Increasing CD58 expression contributes to alleviate ICB resistance	NA	(56, 216)

NA, not available. The presence of NA in the table is due to the lack of information on related studies. ALL, acute lymphoid leukemia; CML, chronic myelocytic leukemia; HL, Hodgkin’s lymphoma; HCC, hepatocellular carcinoma; LAK, lymphokine-activated killing; CTL, cytotoxic T lymphocyte; EMT, epithelial-mesenchymal transition; ICB, immune checkpoint blockade.

## Author Contributions

YZ conceived and drafted the manuscript. QFL revised the manuscript. All authors contributed to the article and approved the submitted version.

## Funding

This work was supported by the National Natural Science Foundation of China (81872501, 81673023, 81272573, and 81502068) and the Beijing Natural Science Foundation (7172177).

## Conflict of Interest

The authors declare that the research was conducted in the absence of any commercial or financial relationships that could be construed as a potential conflict of interest.

## Abbreviations

ADCC, antibody-dependent cellular cytotoxicity; AICD, activation-induced cell death; AITDs, autoimmune thyroid diseases; ALL, acute lymphoid leukemia; AML, acute myelogenous leukemia; APC, antigen-presenting cell; ATRA, all-trans retinoic acid; BL, Burkitt’s Lymphoma; CHO, Chinese hamster ovary; CIA, collagen-induced arthritis; CML, chronic myelocytic leukemia; CMV, cytomegalovirus; CNS, central nervous system; CRC, colorectal cancer; CTL, cytotoxic T lymphocyte; DC, dendritic cell; DLBCL, diffuse large B cell lymphoma; E, erythrocyte; EBV, Epstein-Barr virus; EC, endothelial cell; Fas-L, Fas ligation; GC, gastric cancer; GPI, glycosylphosphatidylinositol; HCC, hepatocellular carcinoma; HL, Hodgkin’s lymphoma; HRS, Reed/Sternberg; IBD, inflammatory bowel disease; ICB, immune checkpoint blockade; IEC, intestinal epithelial cell; IEL, intraepithelial lymphocyte; IS, immunological synapse; LAK, lymphokine-activated killing; LFA-3, lymphocyte-function antigen 3; mAb, monoclonal antibody; MHC, major histocompatibility complex; MS, multiple sclerosis; NF, nuclear factor; NMO, neuromyelitis optica; PHA, phytohemagglutinin; PI-PLC, phosphatidylinositol-specific phospholipase C; PNH, paroxysmal nocturnal hemoglobinuria; RA, rheumatoid arthritis; rv-CD58, recombinant virus bearing CD58; sCD58, soluble CD58; SMAC, supramolecular activation complex; SNP, single-nucleotide polymorphism; SRBC, sheep red blood cell; T11TS, T11 target structure; TCR, T cell receptor; Tregs, T regulatory cells; UV, ultraviolet.

## Glossary

**Table:**

ADCC	antibody-dependent cellular cytotoxicity
AICD	activation-induced cell death
AITDs	autoimmune thyroid diseases
ALL	acute lymphoid leukemia
AML	acute myelogenous leukemia
APC	antigen-presenting cell
ATRA	all-trans retinoic acid
BL	Burkitt’s lymphoma
CHO	Chinese hamster ovary
CIA	collagen-induced arthritis
CML	chronic myelocytic leukemia
CMV	cytomegalovirus
CNS	central nervous system
CRC	colorectal cancer
CTL	cytotoxic T lymphocyte
DC	dendritic cell
DLBCL	diffuse large B cell lymphoma
E	erythrocyte
EBV	Epstein-Barr virus
EC	endothelial cell
Fas-L	Fas ligation
GC	gastric cancer
GPI	glycosylphosphatidylinositol
HCC	hepatocellular carcinoma
HL	Hodgkin’s lymphoma
HRS	Reed/Sternberg
IBD	inflammatory bowel disease
ICB	immune checkpoint blockade
IEC	intestinal epithelial cell
IEL	intraepithelial lymphocyte
IS	immunological synapse
LAK	lymphokine-activated killing
LFA-3	lymphocyte-function antigen 3
mAb	monoclonal antibody
MHC	major histocompatibility complex
MS	multiple sclerosis
NF	nuclear factor
NMO	neuromyelitis optica
PHA	phytohemagglutinin
PI-PLC	phosphatidylinositol-specific phospholipase C
PNH	paroxysmal nocturnal hemoglobinuria
RA	rheumatoid arthritis
rv-CD58	recombinant virus bearing CD58
sCD58	soluble CD58
SMAC	supramolecular activation complex
SNP	single-nucleotide polymorphism
SRBC	sheep red blood cell
T11TS	T11 target structure
TCR	T cell receptor
Tregs	T regulatory cells
UV	ultraviolet.
